# Lowering Cardiovascular Disease Risk for People with Severe Mental Illnesses in Primary Care: A Focus Group Study

**DOI:** 10.1371/journal.pone.0136603

**Published:** 2015-08-28

**Authors:** Alexandra Burton, David Osborn, Lou Atkins, Susan Michie, Ben Gray, Fiona Stevenson, Hazel Gilbert, Kate Walters

**Affiliations:** 1 Epidemiology and Applied Clinical Research Department, Division of Psychiatry, Faculty of Brain Sciences, University College London, London, United Kingdom; 2 Centre for Behaviour Change, Department of Clinical, Educational and Health Psychology, Division of Psychology and Language Sciences, Faculty of Brain Sciences, University College London, London, United Kingdom; 3 The McPin Foundation, London, United Kingdom; 4 Department of Primary Care and Population Health, Institute of Epidemiology and Health Care, Faculty of Population Health Sciences, University College London, London, United Kingdom; University of Stirling, UNITED KINGDOM

## Abstract

**Background:**

People with severe mental illnesses die early from cardiovascular disease. Evidence is lacking regarding effective primary care based interventions to tackle this problem.

**Aim:**

To identify current procedures for, barriers to, and facilitators of the delivery of primary care based interventions for lowering cardiovascular risk for people with severe mental illnesses.

**Method:**

75 GPs, practice nurses, service users, community mental health staff and carers in UK GP practice or community mental health settings were interviewed in 14 focus groups which were audio-recorded, transcribed and analysed using Framework Analysis.

**Results:**

Five barriers to delivering primary care based interventions for lowering cardiovascular risk in people with severe mental illnesses were identified by the groups: negative perceptions of people with severe mental illnesses amongst some health professionals, difficulties accessing GP and community-based services, difficulties in managing a healthy lifestyle, not attending appointments, and a lack of awareness of increased cardiovascular risk in people with severe mental illnesses by some health professionals. Identified facilitators included involving supportive others, improving patient engagement with services, continuity of care, providing positive feedback in consultations and goal setting.

**Conclusion:**

We identified a range of factors which can be incorporated in to the design, delivery and evaluation of services to reduce cardiovascular risk for people with severe mental illnesses in primary care. The next step is determining the clinical and cost effectiveness of primary care based interventions for lowering cardiovascular risk in people with severe mental illnesses, and evaluating the most important components of such interventions.

## Introduction

Compared to the general population, people with severe mental illnesses (SMI) are at an increased risk of cardiovascular disease (CVD) from a younger age and are up to three times more likely to die from CVD [1]. The disparity between the physical health of people with SMI and that of the general population has been the subject of policy and campaigns for over 10 years, with the aim to reduce premature mortality in people with SMI and improve the provision of health care to people with SMI [2–6]. One key debate has focused on who is responsible for the physical health of people with SMI. In the United Kingdom (UK), national guidelines [2] maintain that primary care should take the lead in monitoring the physical health of people with schizophrenia or psychosis.

Despite the evidence, and policy drive to improve the physical health of people with SMI, there is a low uptake of physical health checks among this population [7, 8]. Training nurses to screen for cardiovascular risk factors in people with SMI has modest success in increasing the uptake of physical health checks for this population in primary care [9–10]. A few studies have explored barriers preventing people with SMI from accessing primary care services for a range of health problems [11–14]. Health professionals have cited a lack of understanding of SMI and antipsychotic side effects as barriers to providing optimal care [11], while service users report practical problems such as travel difficulties and fear of attending appointments on their own as deterrents to seeking help from their GP [12, 14]. While evidence for interventions which target multiple CVD risk factors is currently lacking [15], there have been a number of trials which support the effectiveness of interventions to manage weight gain [16–17] and smoking cessation [18] for people with SMI. They have often assessed intensive behavioural interventions which may not be replicable in a primary care setting, or pharmacological treatments which may not be appropriate as a first line prevention strategy; however they demonstrate that healthy lifestyle changes are possible for this group of people.

A systematic review on barriers and facilitators to participation in lifestyle interventions for people with SMI [19] identified two small qualitative studies [20, 21]. Symptoms of mental illness and antipsychotic medication effects were identified as barriers to participation in physical activity [20], while facilitators included one-to-one contact with staff and increased awareness of the positive impact of physical activity gained from participating [20, 21]. Both studies were carried out in psychiatric settings and focused on physical activity alone. No studies have been conducted which identify barriers and facilitators to participating in CVD risk lowering interventions in primary care.

This study aimed to identify current procedures, barriers and facilitators for service users, carers and health professionals to deliver CVD risk lowering interventions for people with SMI in primary care.

## Method

### Study design: Focus group study in primary care and mental health settings

The study was approved by National Research Ethics Committee London–Camden & Islington (11/LO/1475) and research governance approvals were obtained from six institutional review boards covering seven participating NHS trusts in England (NHS Hampshire, NHS Northamptonshire, Northamptonshire Healthcare NHS Foundation Trust, NHS Nottinghamshire, Nottinghamshire Healthcare NHS Foundation Trust, North and Central London Research Consortium (Noclor)).

Only service users who had capacity to consent were invited to take part in the study, as assessed by their GP or mental health worker.

### Sample

Fourteen focus groups with 75 participants were conducted in GP practices or community mental health settings in both urban and rural areas across North London, Northamptonshire, Nottinghamshire and Hampshire, UK between March and August 2012. The groups consisted of between two and eight people (mean group size of five people). We purposively selected participants to represent a range of stakeholders to whom the study aim was relevant including GPs, practice nurses, people with SMI, carers and mental health workers in community mental health teams (CMHTs) (See Table 1).

**Table 1:** Focus Group Composition.

Participants	Number of Groups	Number of participants
Service users	5	25
Practice nurses	3	13
General practitioners (GPs)	2	11
Practice nurses and GPs	1	8^1^
Community mental health staff	2	11
Carers	1	7
**TOTAL**	**14**	**75**

^1^ Comprising of 5 GPs and 3 Practice Nurses

43 health professionals were recruited through the primary care and mental health clinical research networks (PCRN/MHRN) through an email request for expressions of interest to take part in a focus group. Nineteen service users with a diagnosis of schizophrenia, bipolar disorder or schizoaffective disorder were recruited through invitation letters from their GP practice or via a verbal approach from staff working in their GP practice or community mental health service. Six service user experts on the MHRN North and South London hub mailing lists responded to an email request to take part in a focus group at the MHRN West London offices and a group with 7 carers was also arranged through the charity Rethink—Mental Illness.

### Data Collection

Topic guides for focus groups were different for health professionals and for people with SMI/carers. The former aimed to identify the conditions needed for health professionals to effectively lower CVD risk for people with SMI. The latter aimed to elicit information on barriers and facilitators to accessing primary care and ability to perform CVD risk lowering behaviours in people with SMI.

Topic guides were developed by AB (a health services researcher) and reviewed by a multidisciplinary team including health psychologists (LA, HG, SM), a service user researcher (BG) a consultant psychiatrist (DO), a medical sociologist (FS) and a GP (KW). Further feedback was obtained from a Lived Experience Advisory Panel (LEAP), a group of service users and carers who advised the research team on methodological and practical aspects of the study [22].

### Procedure

Twelve of the 14 focus groups were run by a facilitator and co-facilitator who took field notes to ensure that the context of discussions, and other social cues, (e.g., non-verbal agreement, body language, etc.) was preserved. Two groups contained three and two participants so only one researcher facilitated these groups to maintain a balance of participants/researchers. Focus groups lasted approximately one hour, were digitally audio recorded and transcribed verbatim.

Before each focus group, participants completed a written informed consent form with the facilitator, a questionnaire on their demographics, and services users were also asked if they were in contact with mental health services and whether they had any existing CVD risk factors (See S1 and S2 Tables). Participants then introduced themselves, and the facilitator asked the groups to identify current practice and experiences of lowering and managing CVD risk either for themselves or for people with SMI. Once experiences had been elicited and explored, the facilitator then used either the health professional or service user topic guides to stimulate further discussion.

Service users were paid £20 for their time at the end of the group session. Practices were reimbursed for health professional time during practice hours and nurses attending out of practice hours were reimbursed with a £20 gift voucher.

### Data analysis

Data were analysed using the Framework Analysis Method [23]. Analysis proceeded through five stages [24]: 1) familiarisation with the transcripts, 2) identification of a coding framework. 3) application of the coding framework to the data. 4) charting the data and 5) interpretation of themes and associations.

Focus group transcripts were read and corrected with reference to the recordings by the lead author who was present at every group, either as facilitator or co-facilitator (AB). Transcripts and associated field notes were imported into QSR International’s NVivo9 qualitative software package [25] to aid analysis. Three of the authors (LA, AB, BG) independently read and coded the content of the first four transcripts. Coding was compared between authors and any disagreements in coding were discussed until an agreed preliminary coding framework was generated and applied to the remaining transcripts. The final coding framework was developed through an iterative process as further transcripts were analysed and until saturation was reached.

All material was coded in NVivo9 software using the finalised coding framework, with on-going discussion between AB and LA. Codes were then reviewed by LA, AB, KW and DO independently and grouped into three main categories. The original data were synthesised into thematic charts in Microsoft Excel using summarised quotations from the transcripts to illustrate each main category and corresponding themes. The overall interpretation of meaning and explanations were developed, and their implications considered by all authors. Consideration was given to both concordant and discordant views, and both similarities and differences between primary care, CMHT and service user/carer perspectives.

## Results

The analysis was arranged in to the three main categories identified by the a priori study aim: 1) Current procedures for lowering CVD risk for people with SMI in primary care 2) Perceived barriers to delivering interventions for lowering CVD risk for people with SMI in primary care and 3) Perceived facilitators for delivery. Supporting quotations from participants have been chosen to illustrate themes within each category. Categories, themes and a broader selection of supporting quotations can be viewed in S3 Table.

### 1. Current procedures for lowering CVD risk in primary care

#### 1.1 Current CVD risk screening procedures

Most staff and service users reported that physical health checks for people with SMI were being provided to some extent in their GP practice as a result of a UK incentives scheme, the Quality Outcomes Assessment Framework (QOF), which remunerates practices for cardiovascular health screening of people with SMI. This would as ‘best practice’ consist of (i) an annual 20–30 minute appointment with a nurse or healthcare assistant (HCA) to carry out blood tests and clinical measurements (such as BMI and blood pressure) and give lifestyle advice and (ii) a GP consultation to manage medication reviews and discuss test results. Most primary care staff groups acknowledged that although systems for health checks were in place, they did not always conform to this ‘best practice’ model, CVD risk lowering interventions were not being delivered systematically and that not all people with SMI were receiving recommended health checks.

*“We might not do it as well as we could*, *or we might not be doing it as systematically*, *we might not actually be sitting down going*, *'Well*, *that person hasn't come in*,*' it's been done more opportunistically*.*”* (FG10-GP1)

Service users reported mixed experiences of accessing CVD risk lowering interventions in primary care. Some reported having been invited to a CVD health check by the practice, or having check-ups as part of their healthcare for other conditions, such as diabetes.

*“Yeah*, *I’ve had a positive* … *between my CPN and my doctor*, *who sent me a letter recently to say that I could be part of a group that gets reviewed regularly*. *I feel as though I’ve got all the checks in place”* (FG1-Service User5)

Others stated that a health check had only been instigated by the GP in response to them reporting a physical health complaint rather than as a preventative strategy and some reported that they had never received an invitation from their GP to attend a health check.

*“The NICE guidelines recommend that people on Clozaril have a heart check once a year*, *I believe*, *and this has never been done*. *My sister has been on Clozaril for 20-odd years and it's only recently that I've found out that she should*, *and she's been having chest pain*, *that she should have these tests and all these other things for diabetes*, *liver*, *thyroid*. *All these tests should be done and these are just not being done by the GPs*. *They're not even writing a letter to her to say*, *'Come in for these tests*. *They need to be done*.*' It's very poor really*.” (FG9-Carer4)

#### 1.2 Roles and responsibilities for CVD risk lowering intervention delivery

The majority of participants agreed that the implementation of interventions to lower CVD risk in people with SMI was the responsibility of primary care, with service users generally willing to attend a health check and identify healthy lifestyle goals. Health professionals recommended that CVD risk lowering interventions for people with SMI should involve the whole practice as opposed to just the nurse/HCA. It was suggested that GP practices should have some flexibility over delivery; particularly around who delivers what.

*“If you specify who's going to do it*, *then people start going*, *'Oh*, *well*, *I can't do it that way*,*' whereas if you leave it open*, *that's good*. *I mean our practice nurse*, *looking at this*, *probably would do the whole thing and then come to me and say*, *'Oh*, *I've seen such-and-such*, *can I do this*?*' and I'd go*, *'Yeah*, *yeah*, *I know him well*, *that's alright*.*'”* (FG5-GP4)

Some GPs believed that they had a good relationship with service users and felt reluctant to transfer care to the nurse or HCA. Some service users also stated a preference for maintaining contact with their GP as they already had an established relationship, or felt more confident seeing a GP, particularly when presenting with a new health problem and to discuss diagnoses, test results and treatment options.

*“I have more confidence to see a GP than a nurse*. *If I want to enquire about something*, *about a health problem or something*, *it depends what it is*, *but in general I would prefer to see a GP than a nurse*.*”* (FG8-Service User5)

Most service users said they would feel comfortable seeing a nurse for a health check, follow up and monitoring of lifestyle changes, while GPs and practice nurses suggested that a HCA could carry out health checks, smoking cessation, intensive follow up and coordination of care.

*“We have a very good healthcare assistant who does the bloods*, *does some of the health promotion work*, *and then specifically for long term conditions the nurses are involved*.*”* (FG10-GP2)

### 2. Perceived barriers to delivering interventions for lowering CVD risk

Five barriers to lowering CVD risk in people with SMI were identified; 1) negative perceptions of people with SMI, 2) problems with accessing services, 3) low appointment attendance rates, 4) difficulties managing a heathy lifestyle, and 5) a lack of awareness around working with increased CVD risk and SMI.

#### 2.1 Negative perceptions of people with SMI

Some GPs, practice nurses and CMHT staff held negative attitudes towards behaviour change in people with SMI, particularly attitudes towards stopping smoking.Unhealthy behaviours were viewed as a coping mechanism or a “*crutch*” (FG2-Practice Nurse4) for people with SMI.

*“And it may be [smoking]*, *although it's not a very helpful coping mechanism*, *it may be one of their coping mechanisms that they're using to deal with the situation*.*”* (FG14-Occupational Therapist5)

*“It may be for good reasons or it may be from prejudice*, *that you feel*, *'I can't remember the last time I managed to get a patient with really significant mental health problems to stop smoking*.*' Part of me thinks*, *'This person's life is miserable*. *They have a very difficult life and smoking is the one thing they can do*.*"* (FG5-GP5)

In contrast, service users seemed more receptive to improving their physical health if they were given support to do so. Many service users were in agreement with health professionals that achieving a healthy lifestyle was difficult for them; however, all service user groups gave positive examples of trying to achieve better health.

*“Now I'm trying to start looking after myself in terms of eating healthily and not too much*, *and I've started swimming*, *and I do walk a bit*. *But it's trying to build it up slowly*. *I'm not really finding that the weight is coming off particularly easily now*.*”* (FG12-Service User1)

Some service users reported that they had not received support from their GP practice to change unhealthy behaviours, particularly around requests for information on the availability of services and health education.

*“I just saw (the) doctor about my weight*, *for instance*, *and I said about the lithium and I said*, *'I'm trying my best*.*' I've only lost a stone*, *but I said*, *'I'm trying my best*.*' 'We've got bigger women than you*, *so no; we can't really get you into Slimming World or anything like that*.*' So I didn't like that*.*”* (FG12-Service User5)

This may be partly explained by pessimistic attitudes held by some health professionals towards the success of interventions, particularly around weight loss.

*“All the evidence suggests that people who don't take drugs that make you gain weight can't lose weight either*, *so it seems unlikely that patients who are being given drugs that make them gain weight are actually really going to lose weight*.*”* (FG10-GP1)

These attitudes were not universal however, and some GPs reported efforts to approach lifestyle change in people with SMI in the same way as with any other patient.

*“I've seen patients with a severe mental illness who have got to a point where they are prepared to give up smoking*, *and I haven't thought*, *'Well*, *I can't send her to the HCA because she's got schizophrenia*.*'”* (FG10-GP1)

Some primary care health professionals, particularly nurses, described being scared of patients with mental health problems which led to reluctance to offer lifestyle interventions. Some GPs stated that practice nurses in particular lacked confidence in working with this group and that practice nurses may feel apprehensive about seeing people with SMI because they had limited mental health training.

*“Some people find these patients threatening or scary or difficult*. *Depending on the practice and the expertise you've got within it*, *there are bound to be training needs there just in terms of making people feel comfortable with patients who present with more challenging behaviours or difficulties*. *If you're frightened of a patient*, *and lots of professionals who don't work in mental health care*, *and they can be scary*, *and therefore*, *it's much easier not to have the difficult conversation about the fact that*, *yes*, *okay*, *they're on medications*, *but they really could do a bit more to help themselves by losing weight or stopping smoking or not drinking so much*.*”* (FG5-GP5)

GPs own fears on the other hand appeared to be more around the potential workload associated with working pro-actively with people with SMI, who were viewed as more complex and less stable.

*“I think a lot of this fear that you're saying is coming around the idea that they're suddenly going to have a morning full of unstable patients appearing at their doorway*.*”* (FG10-GP1)

Some service users and CMHT staff discussed experiences of ‘diagnostic overshadowing’ where physical health complaints had been dismissed and attributed to the service user’s mental health problem by primary care health professionals.

*“I would just like to be taken seriously when you go*. *I always go and they say things like*, *'Well*, *it could be connected to your mental health or you're feeling anxious* … *' and then you go away and you're back next week because you've still got the same symptoms*. *At the moment*, *I feel well enough to actually challenge that*, *but if you're feeling low*, *you don't have the wherewithal to actually say*, *'It's not my anxiety*. *I know my body*. *I know it's not right*.*”* (FG12-Service User3)

*“I think that sometimes because of having a diagnosis of mental health*, *that's the barrier where it is that*, *'Well*, *it's all in the mind*. *You've got pain*. *It's in the mind*.*’”* (FG11-Community Psychiatric Nurse3)

#### 2.2 Difficulties managing a healthy lifestyle due to antipsychotic medications and symptoms of mental illness

Service users described difficulties losing weight, exercising and eating healthily which they attributed to the medication they took to manage their mental health. Antipsychotics were reported to increase appetite and cause drowsiness by all service user groups, carers and some staff which presented a major barrier to losing weight.

*“Your body doesn’t seem to be able to catch up with what’s happening to it through the medication*. *And there is this*, *as you say*, *this acceptance*, *‘Well*, *that’s the way it is*. *Do you want to feel bad*, *or do you want to be thin*?*’”* (FG7-Service User4)

*“Some of the drugs make you eat more anyway and (make you) sleepy*, *so they put on weight and they don't exercise*.*”* (FG9-Carer5).

All service user groups, carers and some health professional groups identified symptoms of mental illness as a barrier to participating in healthy behaviours. When service users with bipolar disorder experienced high mood, they found it difficult to take in information about health promoting behaviours, while low mood often resulted in a lack of motivation, inactivity and eating foods that were easier to prepare and high in sugar or fat.

“When I’m down I know I’m down because I’m eating a lot of sweets. It makes me feel great for that instant. The trouble is it builds up on the fat, and then it lowers the mood because you’re like that. You end up in a downward spiral, and it’s very hard to get up. Because that becomes part of your normal day, you can’t see what’s happening to you.” (FG4-Service User1)

Some primary care health professionals perceived that it would be inappropriate to carry out health checks and offer lifestyles interventions to service users who were mentally unwell and that lifestyle interventions would only be effective if the service user had stable mental health.

“Sometimes….they will come in and it will be a disaster. Something has happened and actually it's about sorting out the mental health issue that day, so there's just no time to do the blood pressure and talk about smoking. Actually, it's not appropriate, if they're hallucinating or having a crisis.” (FG5-GP1)

It was widely recognised that lifestyle advice might need to be more tailored for people with SMI, because of additional problems with symptoms, motivation and medication.

“Usually they’re aware they’re overweight or they want to exercise more, they want to feel better usually. And often their mental health problem is one extra thing. I mean, there’s people without mental health problems who struggle to exercise and lose weight, and they’ve got motivation–well, they’ve got some, but they’ve got even less motivation, and the drugs often will cause the problems, cause them to be sleepy, cause them to feel sluggish, constipated, dizzy, don’t want to be seen out because of the way they walk in a strange way. So it’s just finding different ways, isn’t it, to get them to move more and do more.” (FG2-Practice Nurse2)

#### 2.3. Difficulties accessing services

Although service users did not experience major difficulties in accessing their GP or practice nurse, concerns were raised by some service users and carers about the lack of proactive invitation for health checks from the GP practice. Some service users and carers mentioned having to actively request physical health checks.

“Where they’re perhaps not very good is that I tend to be the one who has to remind (the GP practice) about my yearly MOT, as I like to call it. Now, I don’t think that’s very good.” (FG1-Service User2)

Other practical barriers to accessing GP services identified by service users included: being offered only morning appointments, experiencing long waits in the GP practice which caused stress, not being able to get through to the practice on the telephone to make an appointment, long waits to see dedicated GPs rather than an unfamiliar GP and being asked by receptionists to disclose confidential health problems. Primary care staff also reported that they could not carry out a comprehensive assessment of CVD risk with people with SMI due to limited consultation time to address multiple problems.

*“And I think*, *you know how sometimes you only get five minutes for an appointment*, *maybe if you had 15 minutes*, *something like that*, *rather than just in and out and you have to take a list in with you because you know you're not going to have time to talk about it*.*”* (FG12-Service User3)

“And if you could do one thing in that appointment, you had to choose the one thing, because you knew you weren’t going to get the whole … you’re not going to be able to work through your template. You think, ‘Well, if I can get this blood pressure today, or I can do one thing today, then I’ve got somewhere.’” (FG2-PN2)

Service users, GPs and CMHT staff also reported a lack of incentivised or free services for people with SMI, as a potential deterrent to being physically active.

*“One of our patients I was trying to refer for a local scheme*, *actually the funding has been cut*, *for weight reduction*.*”* (FG5-GP3)

#### 2.4. Non-attendance at appointments

Primary care health professionals identified a failure to attend appointments as the main barrier to delivering CVD risk lowering interventions to people with SMI. Service users were generally invited by letter to attend a physical health check. Once they had failed to attend three consecutive appointments, there was often no further follow up. This was seen as a limitation by many; however it was felt that limitations were needed for those who were unwilling to engage with services so that this time could be spent seeing other patients.

*“Their failure to attend is so high that they do slip through the system*. *Because you’re sending out a letter*, *aren’t you*, *and a lot of them just don’t attend*.*”* (FG6-Practice Nurse6)

*“Being invited to the GP surgery*, *I mean that sounds lovely*, *but a lot of people would just put that letter in the bin and think*, *'Oh*, *there's nothing wrong with me*.*”* (FG9-Carer4)

Some staff felt that it was also important to respect individual choice if service users did not want to attend the physical health check.

*“If they’re got issues*, *then you can lift the phone up*, *but sometimes people do make a conscious decision if they’ve got severe mental health issues that they don't want to attend their appointments*. *And they are quite within their rights to do that really*.*”* (FG13-Practice Nurse3)

Some staff also stated that younger people were less likely to attend appointments because they may not think that CVD risk lowering interventions were appropriate for them.

*“Some of the younger people on the SMI register are wanting to live very normal lives and they’re working or at uni*. *I can think of a couple of ours that are frequent DNA-ers because they’re busy and they’re trying to live the same as everybody else …… They kind of normalise*. *They don’t see it as a physical or a potential impact on their physical health*.*”* (FG6-Practice Nurse2)

In contrast to staff views, which predominantly attributed non-attendance at appointments to individual patient factors, some service users cited difficulties accessing their GP as the main barrier to attending appointments.

*“It can’t be the next day*, *or even that week often*. *They’re (the GP) generally away*, *and that’s what makes it quite difficult as well*. *It can be quite stressful*. *And then you might not come*, *because you’ve been told you can’t see him for a couple of weeks*. *That can put you off*, *just even picking the phone up*.*”* (FG7-Service User5)

Some service users, carers and health professionals acknowledged that there was limited scope for following up patients who did not attend primary care appointments.

*“My son was almost going to be taken off the register*. *There is no follow up*, *so I don't know what kind of monitoring system can be brought in so that these young people who are not accessing the services…*. *if there's any way of getting them to come over*. *I haven't seen any single letter of communication*.*”* (FG9-Carer3)

Time constraints were also cited as a key barrier to following up non-attenders more frequently and assertively.

*“It’s not a problem for patients who are coming to the surgery*, *who come to the screening it’s those patients who are getting missed*. *We do a letter and then we forget about it or you are busy with something else*.*”* (FG3-GP3)

#### 2.5. Lack of awareness around working with increased CVD risk and SMI

Service users had varying levels of awareness about the links between SMI and the development of CVD risk factors. Some were well informed either through advice from health professionals or self-education. Some individuals however were unaware of the increased risk.

*“It’s never been brought up to me that there was a link between mental health and heart disease; until I was telling the psychiatric nurse that I was coming to this*. *She said*, *‘Oh*, *yes*, *you’re more likely to develop heart trouble*,*’ etc*. *And I thought*, *‘Well*, *nobody had* …*’ I mean*, *surely the doctor should pinpoint that*, *and perhaps do an examination once a year to see how your heart is going on and whether you’ve developed diabetes or anything in the meantime*.*”* (FG7-Service User2)

Some primary care health professionals were also unclear about the increased risk, particularly around people with SMI developing CVD risk factors earlier and experiencing CVD events at a younger age than the general population.

*“Do we all know that people are likely to get their cardiovascular event five years earlier*? *If we all knew that and remembered that*, *then actually if we do see somebody opportunistically we might be far more likely to think*, *'Oh*, *better do their blood pressure and their cholesterol*,*' if it's appropriate*. *So I think in amongst that could be sort of general education of GPs and practice nurses in general*.*”* (FG5-GP7)

Health professionals also cited a lack of awareness of evidence for effective behavioural interventions for people with SMI, resulting in a reluctance to offer them during consultations.

*“I do have a lack of confidence in knowing how effective it's going to be*, *or where it's appropriate for people with SMI*. *So as far as I know there isn't very clear evidence of the sorts of behavioural change interventions that are going to work with that group*, *so that leaves me feeling in a bit of a limbo*.*”* (FG10-GP2)

Practice nurses wanted a better understanding of SMI including an insight into the service user’s experience of being unwell, warning signs for people becoming unwell and information on medications, side effects, drug interactions and mental health services.

*“I haven’t really dealt with a lot of severe mental health*. *I think no matter how much you do*, *you always need a bit of education and training to help you go through those things*. *So I wouldn’t say I was overly confident*.*”* (FG2-Practice Nurse3)

### 3. Strategies to facilitate the delivery of interventions for lowering CVD risk

Five key strategies to aid the delivery of CVD risk lowering interventions were identified by health professionals, carers and service users. Some of these strategies were currently used in practice while others were suggested as novel approaches to aid the delivery of interventions to lower CVD risk in people with SMI. The strategies were: 1) Involving supportive others, 2) Improving patient engagement with services, 3) Continuity of care, 4) Providing positive feedback in consultations and 5) Goal-setting.

#### 3.1. Involving supportive others (formal and informal support)

Co-ordinated working and communication between mental health and primary care staff was described as important to successfully reduce CVD risk in people with SMI. Health professionals and service users felt that mental health workers could help service users attend appointments, either by accompanying them to the GP practice, or through access to a named key worker in the event that a service user did not attend, to establish why this might be and to help rearrange appointments.

*“It’s just a thing about the appointment* … *if somebody within secondary mental health services*, *it would be helpful for that to be copied in*, *because that helps keep the whole picture*, *doesn’t it*? *Because then that person can perhaps encourage you to go*.*"* (FG1-Service User2)

*“If the key worker brings them*, *they might be happy to come along*, *so that’s another way of trying to get access to them*." (FG3-GP2)

Carers were seen by service users and mental health professionals as being influential in helping to promote messages about how to improve health, attending appointments with service users and monitoring adherence to treatments.

*“And would you want them (carer) to be involved in this check with you* …?*”* (Facilitator)

*“Definitely*, *come to appointments with me*, *talk to the doctor*, *talk to the nurse*, *be involved in a very real way*.*”* (FG1-Service User3)

*“I think involving carers is important because if they have specific carers*, *maybe a mother that is looking after somebody with diabetes*, *is on a huge amount of medication*, *(it is) about educating or looking at diet*, *symptoms to look for*, *do they have regular screening*? *So they need to be involved in that process*.*”* (FG11-Community Psychiatric Nurse2)

GPs did not discuss the role of informal carers and some practice nurses had negative experiences of involving informal carers in consultations.

*“There was definitely an incident not so long ago*, *a carer that had come in with somebody*, *was like*, *‘Oh*, *no*, *they’ve got to be done*, *they’ve got to be done*,*’ and we’re like*, *‘We can’t force anything*. *If they’re happy for us to do it*, *we’ll do it*, *but we’re not forcing anything on anybody*.*”* (FG2-Practice Nurse1)

Concerns were also raised by two practice nurses around sharing confidential information with carers without first asking the patient.

*“You can't necessarily contact the carer to follow up non-attenders unless you get consent from the client*.*”* (FG13-Practice Nurse3)

#### 3.2. Improving patient engagement with services

Regular monitoring and follow up of service users was suggested as a strategy to increase patient engagement with primary care services, with direct methods such as telephone or text reminders or home visits favoured over letters by service users and some staff.

*“I'd echo [FG5-GP7]'s point about personal knowledge and contact*, *phone calls*. *If I want to get someone in* ….*I'd keep picking up the phone until I got through to them*.*”* (FG5-GP4)

Arranging a double appointment to carry out health checks and to deliver interventions to lower CVD risk was generally thought to be an effective strategy to overcome the barrier of having limited time to consult with people with SMI.

*“I’ve been lucky with my GP; she’s very good at giving you extra time*. *Not allowing you to go before you’ve actually got your… If she finds any mental illness or any terrible illness*, *that it takes some time to actually voice your concerns*.*”* (FG4-Service User1)

Drop in clinics were also suggested as a way of getting patients to attend primary care appointments, particularly in response to patients only being offered early morning appointments by some GP practices.

*“They could do drop-in sessions*, *like how we run our medication clinics*. *Just give people dates*, *'Come on this date between 9*:*00 and 12*:*00*, *just turn up*.*”* (FG11-Community Psychiatric Nurse5)

#### 3.3. Continuity of care

Continuity of care was emphasised as important for building a relationship of trust and familiarity between the service user and practice nurse/GP. It was suggested by health professionals and service users that one practice nurse and/or GP should oversee and deliver interventions to lower CVD risk to people with SMI in each practice in order to build this relationship.

*“I like the fact that it would be probably long term*, *and the fact that you hopefully would see the same person*.*”* (FG12-Service User3)

*“The relationship is important with the patients*, *so people stick to somebody who is responsible for their care in a holistic way*.*”* (FG10-GP2)

#### 3.4. Providing positive feedback in consultations

The importance of promoting and reinforcing healthy behaviours at every consultation in a positive and encouraging way was emphasised. Preaching information and judgemental statements were seen as unhelpful and demotivating.

*“Don't preach*, *tell people facts and give people the right to make that decision*. *It's how you present it because if you say to something*, *'You should be doing this*, *and you should be doing that*,*' uh-oh*. *But*, *'Why don't you try this and why don't you try that*?*”* (FG9-Carer7)

*“In my experience*, *if people don’t turn up*, *it’s not to be judgmental*, *and not to tell them off that they haven’t arrived*, *but just each time they come in say*, *‘Oh*, *you didn’t come last time*. *How about if we can do that today*?*’ or just encourage them to keep coming*.*”* (FG2-Practice Nurse4)

#### 3.5. Goal Setting

Defining small achievable goals in partnership with the service user was suggested as a strategy to change unhealthy behaviours, rather than trying to address multiple CVD risk factors at the same time within a consultation.

*“Targeting just one factor*, *you’ll probably find that having targeted the smoking you’ll find it a lot easier to get active and things like that*. *It’s a knock on effect*. *Whereas if you go in*, *for some people with mental health problems*, *they want to do it all*.*”* (FG4-Service User1)

*“Identifying one key behaviour change*, *it’s really important to give them achievable goals and be realistic*.*”* (FG6-Practice Nurse1)

Breaking down the ultimate outcome in to smaller achievable goals was also suggested as a strategy to achieving a healthy lifestyle:

*“Looking at part goals rather than stopping smoking or getting down to an ideal BMI but making some gains in that direction is useful*.*”*(FG2-GP3).

## Discussion

The results highlight a number of strategies and factors that may prevent and enable people with SMI to access and engage with CVD management in primary care settings. We identified several barriers and facilitators, as well as the need for more systematic approaches to delivering this form of care. The over-riding view was that delivering interventions to lower CVD risk for people with SMI was the responsibility of primary care and professionals felt this could be coordinated by a practice nurse or healthcare assistant while overseen by a GP. This mirrors existing service models for the management of long term conditions such as diabetes in primary care.

Key barriers to lowering CVD risk in people with SMI included difficulties in opportunistic preventative health care, as the focus of consultations are on mental health symptoms rather than on addressing physical health needs, a lack of belief among some health professionals that lifestyle interventions can be effective with this population, and in practice nurses, a lack of training and confidence in working with people who have severe mental illness. Mental health symptoms and their impact on motivation, the adverse effects of anti-psychotic medication, adherence to treatments and attendance at appointments were all cited as factors that might make behaviour change to lower CVD risk more difficult for people with SMI.

A range of strategies were identified to address some of these concerns, including simple practical adjustments to help facilitate attendance and engagement (e.g. afternoon appointments, telephone reminders), identifying and involving supportive others, continuity of care, improving the communication of lifestyle advice within consultations, and breaking down CVD risk management into small achievable goals.

### Relevance to existing clinical policy and research

Our study reinforces findings from previous qualitative work on barriers to accessing primary care services for people with SMI, particularly regarding practical barriers such as difficulties around making appointments and being able to see the same health professional at each consultation [11–14]. Previous qualitative studies with service users in psychiatric settings have identified that symptoms of mental illness and the effects of anti-psychotic medication are barriers to participating in interventions to promote physical activity [20, 21] while one-to-one contact with a health professional was identified as a facilitator. We found that continuity of care and the relationship formed with the same health professional at each appointment were viewed as important drivers.

The negative views held by health professionals regarding behaviour change in patients with SMI are at odds with the evidence. There are positive findings from RCTs and systematic reviews of both smoking and weight reduction in people with SMI [16–18]. Our findings demonstrate that these positive behaviour change findings need to be disseminated more effectively and widely among health professionals who work with people with SMI to try and improve attitudes to routine physical health care.

National guidelines for Schizophrenia [2] suggest that carers should be involved in treatment decisions for people with SMI. This was largely supported by service users and mental health staff in our focus groups but was rarely discussed by GPs. Involving carers in consultations was seen as distracting by some practice nurses and concerns around confidentiality were expressed. Qualitative studies have identified social support as an important motivating factor for people with schizophrenia wanting to lose weight through joint participation in physical activities [26–28] and for people with bipolar disorder who want to stop smoking [29].

Setting small attainable health goals was identified as a potentially useful behaviour change technique to facilitate lifestyle change in people with SMI, and there is evidence to support its effectiveness in other populations, such as for weight loss in overweight and obese adults [30] and in interventions targeting diet and/or physical activity in adults at risk of developing type 2 diabetes [31].

### Implications for practice and further research

This is the first qualitative study which provides an understanding of the challenges and facilitating factors involved in delivering interventions to lower CVD risk for people with SMI in primary care. The study obtained the views of a range of stakeholders from both rural and urban areas making the findings applicable across UK primary care settings and similar health care systems in other countries. The findings may be less transferable to intervention delivery for patients with SMI in non-primary care settings.

It is possible that the service users we spoke to may have been more engaged with health services than most other people with SMI. This may help to explain why health professionals spoke of non-attendance at appointments as one of the biggest barriers to delivering care to patients with SMI, while service users seemed happy to engage with primary care as long as practical barriers were addressed. It is likely that accessing services may be a greater challenge for those who are unwell or not as actively engaged with their GP practice.

While there was acceptance that CVD risk should be managed by primary care health professionals, the perceptions of delivering CVD risk lowering interventions for people with SMI were in part incongruent with current policy recommendations and service user perspectives in the focus groups. For example there is a need to address the misconception among some health professionals that people with SMI do not benefit from CVD risk lowering interventions. An intervention to lower CVD risk in people with SMI should consider targeting health professional attitudes towards behaviour change in people with severe mental illness, as well as provide service users with the confidence and skills to self-manage their physical health. Strategies suggested by this study to encourage self-management included setting and monitoring small achievable lifestyle goals and providing positive feedback, both of which could be incorporated in to primary care consultations.

The study highlighted limited understanding of SMI among some primary care health professionals resulting in a lack of provision of CVD risk lowering interventions, stigmatisation and fear. Mental health training could be incorporated in to the continuing professional development of practice nurses and GPs to improve understanding around the impact of antipsychotic medication and psychiatric symptoms on the physical health of people with SMI and to address fear and stigma.

Some potentially important facilitating factors were identified to promote attendance, motivation and engagement in lifestyle change for people with SMI. For example, involvement of supportive others (e.g. key workers or carers) was highlighted, with the caveat that service users always be asked before approaching supportive others, and is something that could be encouraged more systematically. We can’t however determine from this research the extent to which service users have a supportive other who could take on this role, and whether they would want their mental health worker or carer involved in CVD risk lowering interventions.

The facilitators and barriers provide a range of domains which clinical managers, policy makes and researchers can address in efforts to design, deliver and evaluate CVD risk reduction in their localities. We have incorporated the findings into a trial of an intervention in UK primary care. This ongoing trial is evaluating a nurse-led intervention to manage CVD risk in general practices across England [32]

## Conclusion

Findings from these focus groups suggest that while systems for health checks in primary care are in place for people with SMI, on-going CVD risk management is not being carried out systematically. The findings suggest that both service users and health professionals support the delivery of CVD risk lowering interventions in primary care; however barriers identified by this study and previous research need to be considered when designing new interventions, to ensure effective implementation in practice and maximum take up from service users. Our study presents a focused exploration of existing practice, barriers and facilitators to delivering CVD risk lowering interventions in primary care and should be used to inform the implementation of existing interventions to lower CVD risk, adapted to meet the needs of people with SMI and primary care. An evaluation of the effectiveness of such interventions in primary care settings is required.

## Supporting Information

S1 Table Participant characteristics: all groups. (PDF)

S2 Table Participant characteristics: service user and carer groups. (PDF)

S3 Table Supplementary quotations to illustrate the barriers and facilitators sub themes. (PDF)
