# Supplementary material for: Lowering Cardiovascular Disease Risk for People with Severe Mental Illnesses in Primary Care: A Focus Group Study
Source: PLoS One. 2015 Aug 28;10(8):e0136603. doi: 10.1371/journal.pone.0136603 (PMC4552729; doi:10.1371/journal.pone.0136603)
Supplement: S1 Table — (PDF) [file pone.0136603.s001.pdf]

S1 Table. Participant characteristics: all groups

Group	Service users (N = 25)	Carers (N=7)	Practice Nurses (N=16)	General Practitioners (N=16)	CMHT staff (N=11)
**Mean Age (Range)**	49 (31-68)	55 (32-66)	52 (42-68)	46 (28-60)	44 (29-51)
**Gender:**					
Female	16 (64%)	7 (100%)	16 (100%)	11 (69%)	9 (82%)
Male	9 (36%)	0%	0%	5 (31%)	2 (18%)
**Ethnicity**					
White British	16 (64%)	6 (86%)	12 (75%)	11 (69%)	10 (91%)
Black African	3 (12%)	1 (14%)	0 (0%)	0 (0%)	1 (9%)
Black Caribbean	1 (4%)	0 (0%)	0 (0%)	0 (0%)	0 (0%)
Indian	1 (4%)	0 (0%)	1 (6%)	3 (19%)	0 (0%)
Mixed Asian and African	0 (0%)	0 (0%)	0 (0%)	1 (6%)	0 (0%)
Asian Other	2 (8%)	0 (0%)	0 (0%)	0 (0%)	0 (0%)
White Irish	1 (4%)	0 (0%)	0 (0%)	0 (0%)	0 (0%)
White Other	1 (4%)	0 (0%)	3 (19%)	1 (6%)	0 (0%)
