# Supplementary material for: Lowering Cardiovascular Disease Risk for People with Severe Mental Illnesses in Primary Care: A Focus Group Study
Source: PLoS One. 2015 Aug 28;10(8):e0136603. doi: 10.1371/journal.pone.0136603 (PMC4552729; doi:10.1371/journal.pone.0136603)
Supplement: S2 Table — (PDF) [file pone.0136603.s002.pdf]

S2 Table. Participant characteristics: service user and carer groups

Group	Service users (N=25)	Carers (N=7)
**Employment status**		
Unemployed	14 (56%)	1 (14%)
Retired	5 (20%)	2 (29%)
Part time employment	2 (8%)	1 (14%)
Full time employment	2 (8%)	1 (14%)
Voluntary work	2 (8%)	0 (0%)
No response	0 (0%)	2 (29%)
**Marital status**		
Married or in a relationship	9 (36%)	4 (57%)
Single	13 (52%)	0 (0%)
Divorced	3 (12%)	1 (14%)
No response	0 (0%)	2 (29%)
**Self-reported diagnosis**		
Schizophrenia	7 (28%)	4 (57%)
Schizotypal disorder	1 (4%)	0 (0%)
Bipolar affective disorder	17 (68%)	3 (43%)
**In contact with mental health services?**		
Yes	19 (76%)	5 (72%)
No	4 (16%)	1 (14%)
Don't know	2 (8%)	1 (14%)
**Self-reported CVD Risk factors:**		
No reported CVD risk factors	12 (48%)	4 (57%)
1+ CVD risk factor	13 (52%)	3 (43%)
