# Supplementary material for: Lowering Cardiovascular Disease Risk for People with Severe Mental Illnesses in Primary Care: A Focus Group Study
Source: PLoS One. 2015 Aug 28;10(8):e0136603. doi: 10.1371/journal.pone.0136603 (PMC4552729; doi:10.1371/journal.pone.0136603)
Supplement: S3 Table — (PDF) [file pone.0136603.s003.pdf]

S3 Table: Supplementary quotations to illustrate the barriers and facilitators sub themes

**Category/*Sub theme***	**Supporting Quotations**
**Perceived barriers to managing CVD risk in SMI patients**	
Negative perceptions of people with SMI	***Negative attitudes towards behaviour change in SMI***<br>*I think the difference possibly is with people with severe mental health issues, is things are often much more difficult for them, because if we take smoking as an example, even for people that haven't got problems with mental health, it's a crutch. And it's sometimes a step too far for anybody. If you're having problems just coping with day to day living, it can be difficult (FG2-Practice nurse4)*<br><br>*I just saw (the) doctor about my weight, for instance, and I said about the lithium and I said, 'I'm trying my best.' I've only lost a stone, but I said, 'I'm trying my best.' 'We've got bigger women than you, so no; we can't really get you into Slimming World or anything like that.' So I didn't like that (FG12-Service user5)*<br><br>*When you start the conversation with patients with mental illness smoking is a pleasure to them. There aren't many pleasures; their social world has often fallen apart, support structures are limited. I back off from pushing that. (FG10-GP1)*<br><br>*I think the amount of people who are going to actually change behaviour or change probably isn't that high. It doesn't mean that you shouldn't try" (FG2-GP3)*<br><br>*A lot of people with psychoses have a lot of paranoia, they have a lot of panic attacks, and the one thing that helps them relax is smoking and if you tell them to remove that, you make their condition worse, quite often (FG5-GP3)*<br><br>*I was quite surprised how well some of my patients took to the idea of being called in for an annual review. I suppose I came at it with that idea of, 'They'll be a bit resistant and the last thing they want is to be nagged about their smoking, and it's time consuming... I prejudged how they would respond" "It may be for good reasons or it may be from prejudice, that you feel, 'I can't remember the last time I managed to get a patient with really significant mental health problems to stop smoking.' Part of me thinks, 'This person's life is miserable. They have a very difficult life and smoking is the one thing they can do." "I think there's a sense of hopelessness and if that's not justified because there's good evidence that actually you improve people's lives, then tell us that and disseminate it widely (FG5-GP5)*

Category/*Sub theme*	Supporting Quotations
**Perceived barriers to managing CVD risk in SMI patients**	
Negative perceptions of people with SMI	*They're having bloods, they know what they're coming for, they're happy with it, and they know what's happening. But to tell them to change their lifestyle that's another story (FG10-Practice nurse8)*<br><br>***Fear and Stigma***<br><br>*Because of the stigma of mental health, I find that when some of my clients have perhaps gone to see the practice nurse or doctor, it seems like they've tried to spend minimal time with them as possible. 'Oh, 'They've got a mental health problem.' Because last time one of my... I've got one female client actually, and she went to see the practice nurse and I said, 'What did she do?' and she said, 'Oh, she took my blood pressure and then said I was fine and sent me out.' She didn't really spend any time with her at all. Didn't even liaise with me, didn't send a letter to our doctors or anything, didn't explain what had happened and why. It just seems to be like this thing where they don't want to spend any time (FG11-Community Psychiatric Nurse4)*<br><br>*I think the reason why you've had such a poor turnout from practice nurses, and why I've been thrown here from my practice, is because the practice nurses just feel totally uncomfortable with mental health issues. I get a lot of support from a particular GP who works with me (FG10-Practice Nurse4)*<br><br>*People are still a bit frightened sometimes, with mental health, and particularly chronic mental health. (FG6-Practice nurse2)*<br><br>*I wouldn't underestimate how much fear there is. It's not just about giving information; it's about trying to unpick what people's preconceptions are and what concerns them so much (FG10-GP2)*<br><br>*People really respond to the physical things I've had, there's a response of sympathy and care, but with eccentric behaviour there's a fear (FG7-Service user3)*<br><br>*I believe, correct me if I'm wrong, but most of the nurses do not want to deal with people with a severe mental illness. (FG3-GP2)*<br><br>*They might get the wrong idea and you get all of us having a big mentally ill… we see mentally ill... like drug addicts and all these people coming into the services. We don't want that to happen more (FG3-GP3)*<br><br>*If you ring the surgery and make an appointment, like I sometimes do for my husband, and I said, 'I need an appointment,' a heart*

Category/*Sub theme*	Supporting Quotations
**Perceived barriers to managing CVD risk in SMI patients**	
Negative perceptions of people with SMI	*patient, they would give straightaway, but if you told them about the mental illness, they say, 'You might have to go to the hospital.' That's wrong (FG9-Carer5)*<br><br>*They're so used to being a bit guarded and wary of people judging them with a diagnosis on their notes, and actually I know a couple of people here that still may go, 'Uhh, what do you do with a schizophrenic?' or, 'Uhh, what do we say?' That sort of thing, because people are still a bit frightened sometimes, with mental health, and particularly chronic mental health (FG6-Practice nurse2)*<br><br>*Some people find these patients threatening or scary or difficult. Depending on the practice and the expertise you've got within it, there are bound to be training needs there just in terms of making people feel comfortable with patients who present with more challenging behaviours or difficulties. If you're frightened of a patient, and lots of professionals who don't work in mental health are, and they can be scary, and therefore, it's much easier not to have the difficult conversation about the fact that, yes, okay, they're on medications, but they really could do a bit more to help themselves by losing weight or stopping smoking or not drinking so much (FG5-GP5)*<br><br>***Diagnostic Overshadowing***<br><br>*But I think that sometimes because of having a diagnosis of mental health, that's the barrier where it is that, 'Well, it's all in the mind. You've got pain. It's in the mind" (FG11-Community psychiatric nurse3)*<br><br>*I've had my GP a long time, he knows about my mental health. But if ever I come with a physical ailment, he's very dismissive. 'Are you sure you're feeling this? Are there any other explanations?' He doesn't ever take me seriously.....generally if you've got a mental health problem, you come in with a physical ailment – generally, unless you've got a pretty good doctor, pretty good GP, you're not listened to (FG1-Service user3)*<br><br>*I would just like to be taken seriously when you go. I always go and they say things like, 'Well, it could be connected to your mental health or you're feeling anxious... you're doing this, you're doing that,' and then you go away and you're back next week because you've still got the same symptoms. At the moment, I feel well enough to actually challenge that, but if you're feeling low, like people have said, you don't have the wherewithal to actually say, 'It's not my anxiety. I know my body. I know it's not right' (FG12-Service user3)*

Category/*Sub theme*	Supporting Quotations
**Perceived barriers to managing CVD risk in SMI patients**	
Negative perceptions of people with SMI	*But the GP, instead of going, 'Right, we better follow this up. We'd better do an ECG, we'd better check what it is,' rang the consultant... no, rang here, sorry, rang here and said, 'This lady is under mental health. You'd better sort her out,' instead of saying, 'This lady has got a physical problem, we'll check it, but can we liaise with you because of her medication?' It was pushed straight back, 'This is mental health' (FG11-Community psychiatric nurse3)*
Difficulties managing a healthy lifestyle	***Antipsychotic Medications***<br><br>*The initial one I was on, chlorpromazine, one of the basic things is stimulating appetite, and a lot of them do that..... And people aren't daft, they look in the mirror when they're on a high medication sedative, and they know they can't function, yet they would like to, and they've got the brain to, but they're just thwarted because of the medication (FG1-Service user1)*<br><br>*Your body doesn't seem to be able to catch up with what's happening to it through the medication. And there is this, as you say, this acceptance, 'Well, that's the way it is. Do you want to feel bad, or do you want to be thin?' (FG7-Service User4)*<br><br>*Some of the drugs make you eat more anyway and (make you) sleepy, so they put on weight and they don't exercise (FG9-Carer5)*<br><br>*You've got to reduce the medication and manage it better to see whether there is a change in the blood sugar, or blood pressure. I think those two have got to work together in medication control. You can't just leave the medication going; it won't make any impact on the diabetes or high blood pressure because it will just get worse. They've got to manage it together (FG9-Carer2)*<br><br>*The drugs they're on, often they can put on weight, it can make them sluggish, it affects their diabetes. It has a knock-on effect about everything (FG2-Practice Nurse4)*<br><br>*It does feel like it's the drugs we give them that increase their risk, so we can send them to all the places in the world, but actually they're not going to lose weight. They're not going to lose weight as long as you keep giving them the drugs that are controlling their mental health condition.....so it seems unlikely that patients who are being given drugs that make them gain weight are actually really going to lose weight (FG10-GP1)*<br><br>***Symptoms of Mental Illness***<br><br>*When I'm down I know I'm down because I'm eating a lot of sweets. I eat a lot of sweet stuff. It makes me feel great for that instant. The trouble is it builds up on the fat, and then it lowers the mood because you're like that. You end up in a downward*

Category/*Sub theme*	Supporting Quotations
**Perceived barriers to managing CVD risk in SMI patients**	
Difficulties managing a healthy lifestyle	*spiral, and it's very hard to get up (FG4-Service user1)*<br><br>*Since I've been taking mood stabilisers, which probably is about two years, I've put about two stone on. But I think part of that was because I was feeling low a lot of that time and not doing much and eating unhealthy foods, so I wasn't really caring about my health very much (FG12-Service user1)*<br><br>*I find getting exercise quite difficult now, because going out causes me such anxiety (FG7-Service user5)*<br><br>*So it's understanding that people who suffer from mental illness can be very chaotic and that can include missing appointments, not because they don't want to come. Some people do want to go to the appointments, but for whatever reason they end up not going (FG11-Community psychiatric nurse5)*<br><br>*If people's mental health is in a good state they will be more willing to take care of their bodies. Whereas if their mental health is in a poor state, then that will not be important to them. Also if their mental health is in a good state they will be able to engage in behaviours which are positive, as opposed to behaviours which damage their health. So perhaps they'll go out and make friends, as opposed to sitting at home and smoking because they haven't got anything else to do and to kind of treat the pain of that. So the key to a person with mental health problems physical health is often their mental health (FG10-GP7)*<br><br>*A lot of patients unless they're well controlled, your consultation is quite busy with managing other stuff going on (FG10-GP1)*<br><br>*I think there's only a point in attempting to do physical health interventions if they actually have the mental space to accommodate those (FG10-GP7)*<br><br>*A patient I've got who is diabetic, he's only young, he's already lost a leg, and I know that every time he goes into a psychotic state I have to start again with his diabetes. It's so difficult (FG13-Practice nurse3)*<br><br>*There are days when they are so low that even getting out of bed is an effort. Their eating pattern is so haywire. Sleeping pattern is absolutely chaotic. There will be days when they're so high, there is such rapidity of movement, that you just want to say to the person, 'Stop, think, slow down,' but it cannot happen because it's the mental state that the person is going through. You have to be able to know that. So where is that awareness, 'Oh, I'm putting on weight. I should think about diabetes and go? (FG9-Carer2)*

Category/*Sub theme*	Supporting Quotations
**Perceived barriers to managing CVD risk in SMI patients**	
Difficulties accessing services	*And I think, you know how sometimes you only get five minutes for an appointment, maybe if you had 15 minutes, something like that, rather than just in and out and you have to take a list in with you because you know you're not going to have time to talk about it (FG12-Service user3)*<br><br>*And if you could do one thing in that appointment, you had to choose the one thing, because you knew you weren't going to get the whole... you're not going to be able to work through your template. You think, 'Well, if I can get this blood pressure today, or I can do one thing today, then I've got somewhere (FG2-Practice nurse2)*<br><br>*Sometimes if I ring if it's mid to late morning they may not have any emergency appointments. I phoned up and I wasn't feeling well and they said, 'Oh, you'd better go to the walk-in centre then.' I said, 'Well, I don't feel well enough to go there,' and they said, 'Well, go to A&E.' I said, 'Well, that's even further and I don't know how to get there.' They said, 'Get a taxi.' I said "I can't afford a taxi,' because it's £15 from my house to A&E. So I just put the phone down and gave up and I didn't go anywhere. It turned out, a week later, that I'd had a mini stroke. I was quite upset about that (FG12-Service user3)*<br><br>*Sometimes it's about making it easier for them to engage. I think a lot of people, they don't necessarily make the phone calls because they've got this on the other end of the phone, 'Press this for this, and this for that,' and their anxiety becomes... and they can be sat on the phone for 20 minutes and they give up. It's upset them too much that they don't want to carry on with it. So it stops them from making that initial call (FG14-Community development worker6)*<br><br>*Accessing own GP can be difficult. It can't be the next day, or even that week often. They're generally away, and that's what makes it quite difficult as well. It can be quite stressful. And then you might not come, because you've been told you can't see him for a couple of weeks. That can put you off, just even picking the phone up (FG7-Service user5)*<br><br>*And even the gym on referral, it's only for ten or twelve weeks and they still have to pay... it's about £3 a session, which is quite a lot of money for some individuals (FG14-Occupational therapist5)*<br><br>*Because one of the deterrents is often that you perhaps want to get fit – and I'm very fit, I'm fortunate, I love my walking. But if I wasn't, I would feel, 'Oh, heck, if I want to go to the gym, that will cost me quite a lot of money,' even going to the swimming pool. So although you get a little bit of a reduction, it's not as big a reduction as it used to be (FG1-Service user2)*

Category/*Sub theme*	Supporting Quotations
**Perceived barriers to managing CVD risk in SMI patients**	
Difficulties accessing services	*One of our patients I was trying to refer for a local scheme, that actually the funding has been cut, for weight reduction (FG5-GP3)*
Non-attendance at appointments	*The biggest problem we have is non-attenders trying to get them to see us. I mean they don't come easily and we waste a lot of appointments, we book them and they don't come. Three times I will send a form for an MH14, a cholesterol thing, three times I have to print the forms and the person we are trying to contact, they don't simply come (FG3-GP4)*<br><br>*As well is their failure to attend they do slip through the system. Because you're sending out a letter, aren't you, and a lot of them just don't attend. Unless you do see them for something else, and while they're there you try and get it all done. But specifically attending for what they need to attend for, their reviews and stuff, the failure rate is quite high (FG6-Practice nurse6)*<br><br>*Being invited to the GP surgery, I mean that sounds lovely, but a lot of people would just put that letter in the bin and think, 'Oh, there's nothing wrong with me (FG9-Carer4)*<br><br>*And also if you missed one appointment, you know, some people might be cross at that saying, 'Well, we're not doing it again because you missed the first appointment (FG14 Community psychiatric nurse2)*<br><br>*I think the real difficulty is for the people who don't engage. If they're happy to come in and have blood tests fine, but in my experience some people don't want intervention, they don't want blood tests, and then having practice nurses do it would be difficult because they don't go out of the surgery. You may have to involve district nurses, because I think it's the people that don't come probably who are most needing to (FG5-GP7)*<br><br>*You spend your life chasing patients (FG13-Practice nurse2)*<br><br>*The trouble is with things that we found, if people's appointments are too far in the future, they tend to forget them. The ones that just come for six monthly or yearly monitoring, because they're doing okay, often forget (FG14-Psychiatrist3)*<br><br>*Some of the younger people on the SMI register are wanting to live very normal lives and they're working or at uni. I can think of a couple of ours that are frequent DNA-ers because they're busy and they're trying to live the same as everybody else (FG6-Practice nurse5)*

Category/*Sub theme*	Supporting Quotations
**Perceived barriers to managing CVD risk in SMI patients**	
Non-attendance at appointments	*These patients are very time consuming, they don't keep appointments and if they don't turn up for a practice nurse appointment of 20 mins or a 15 min slot, it's wasted (FG3-GP1)*<br><br>*I think the only main problem would be following up the non-attenders and being persistent in trying to get them to come in (FG6-Practice nurse3)*<br><br>*Some patients do not want to engage with you at all, so that's where the difficult comes along because they point blank refuse any kind of contact, they don't even allow you to go inside and in those patients what happens is they are not even followed by the psychiatric side of things (FG3-GP2)*<br><br>*I guess the other problem is if they're not engaging with us, are they definitely going to the mental health services because there are plenty of clinics where very few patients turn up for their mental health reviews at psychiatry. I think there is a problem of disengagement in all areas (FG3-GP3)*
Lack of awareness around working with increased CVD risk and SMI	*That's education though, isn't it, for practice nurses as well, because I think a lot of people probably don't realise the link between increased (CVD) risk and mental health, do they? (FG6-Practice nurse3)*<br><br>*I do have a lack of confidence in knowing how effective it's going to be, or where it's appropriate for people with SMI. So as far as I know there isn't very clear evidence of the sorts of behavioural change interventions that are going to work with that group, so that leaves me feeling in a bit of a limbo. So that's my gap more than the screening, actually (FG10-GP2)*<br><br>*Do we all know that people are likely to get their cardiovascular event five years earlier? If we all knew that and remembered that, then actually if we do see somebody opportunistically we might be far more likely to think, 'Oh, better do their blood pressure and their cholesterol,' if it's appropriate. So I think in amongst that could be sort of general education of GPs and practice nurses in general, quite apart from setting up a scheme really (FG5-GP7)*<br><br>*Maybe they (mental health staff) suffer from what I suffer from, which is that sense of, 'Well, actually, the mental illness side of things is so all pervasive it's as much as we can do to keep up with that.' If the evidence is that that's not the case, then they need education too (FG5-GP5)*

Category/*Sub theme*	Supporting Quotations
**Perceived barriers to managing CVD risk in SMI patients**	
Lack of awareness around working with increased CVD risk and SMI	*I mean, I wouldn't even know what a community psychiatric team would do as far as that's concerned. In one way I would have thought they may have looked at issues with the GP about chronic disease for them (FG6-Practice nurse5)*<br><br>*There's such a lot of education that is needed, and they're probably not getting their health checks. It could just be because nurses aren't informed, they might not have that experience. And that is a big thing about education (FG2-Practice nurse1)*<br>*I haven't really dealt with a lot of severe mental health. I think no matter how much you do, you always need a bit of education and training to help you go through those things. So I wouldn't say I was overly confident (FG2-Practice nurse3)*

Category/*Sub theme*	Supporting Quotations
**Strategies to facilitate CVD risk management in SMI patients**	
Mental health worker and carer involvement	*We do accompany service users to physical health checks if they are unable, or unwilling, or need some support to actually get to the GPs, so we will go with the individuals to ensure that they do go" (FG14-Occupational therapist5)*<br><br>*I think what I would like to see is everybody singing from the same health hymn sheet, and so even if the community psychiatric nurses aren't going to do the work, that they should be reinforcing good stopping smoking, weight, and saying, 'That's really good,' or, 'We'll take you along,' or, 'Have you got your medication?' or whatever, and I think some support from them who are seeing them far more often would be very useful actually (FG5-GP7)*<br><br>*I'm hearing everybody talk, and I'm not hearing anyone say that, 'My partner helps me with my medication,' or, you know. Everyone is talking as a single individual entity, and there's no, 'My mother cares for me,' or, 'My brother cares for me, my partner cares for me.' What about that.....that aspect? (FG1 Service user3)*<br><br>*I try and work out ways they can be motivated to get out and change their lifestyle in other ways. That often requires somebody being attached to them and constantly saying to them, 'Hey, come on out, have a cup of coffee, come on out, let's go for a walk.' Yeah, I think that sort of approach, drip fed, is more likely, at the end of the day, to produce dividends to stop smoking rather than going straight in on stop smoking, because most people won't (FG5-GP3)*

Category/*Sub theme*	Supporting Quotations
**Strategies to facilitate CVD risk management in SMI patients**	
Mental health worker and carer involvement	*We always encourage to stop smoking. They're given the patches to wear and if they're in hospital so we always encourage them (FG14-Psychiatrist3)*<br><br>*(The charity) built up my confidence. And (the support worker) does as well. We go swimming, and badminton on Tuesdays. Thursdays, that's today isn't it? Usually on Thursdays I go over to xxxx Park which is where she lives, there's somewhere we can go swimming, or play tennis, or go for a walk. It's lovely.....It's a lovely place isn't it? We do things like that…. And she challenges me. I challenge her, she challenges me. And that's again through increased level of confidence (FG4-Service user2)*<br><br>*(It is) good to know their social set up and what support they have in place to determine the impact on what you can achieve with the patient (FG6-Practice nurse6)*<br><br>*I know there are issues, but at least we can be gatekeepers and we can say, 'I'm going to take you there. We've been three times, but maybe the fourth time you'll be okay (FG11-Commmunity psychiatric nurse2)*<br><br>*We do accompany service users to physical health checks if they are unable, or unwilling, or need some support to get to the GP... (FG14-Occupational therapist5)*<br><br>*I think involving carers is important because if they have specific carers, maybe a mother that is looking after somebody with diabetes, is on a huge amount of medication, about educating or looking at diet, symptoms to look for, do they have regular screening? So they need to be involved in that process (FG11-Commmunity psychiatric nurse2)*<br><br>*But if you're talking about like (a) partner or something you can't necessarily be contacting them and saying, 'Your husband didn't come for his thing. Unless you get consent from the client" (FG13-Practice nurse1)*<br><br>*If the key worker brings them, they might be happy to come along, so that's another way of trying to get access to them (FG3-GP2)*<br><br>*They need to be able to tell the person they're caring for what's available, like the gym (FG9-Carer6)*<br><br>*If they attend with a carer it's good because you can explain to the carer the detail you want to go into, within the limitations of what you are presented with (FG10-Practice nurse8)*

Category/*Sub theme*	Supporting Quotations
**Strategies to facilitate CVD risk management in SMI patients**	
Mental health worker and carer involvement	*We've got a couple of mental health patients that we rely heavily on their psychi nurses, we liaise with them to bring them in and make changes. It works well, the patient has an understanding, and her liaison nurse knows we'll be contacting them for any major changes (FG6-Practice nurse6)*<br>*My friends take me to the gym every day and that's all what I do, really. I play football with my son and that (FG12-Service user6)*<br><br>*Confidentiality is good in some respects but having someone with you needs to be discussed (FG7-Service user5)*<br><br>*I have a CPN, and she encourages me to go for diabetes tests and stuff like that, so I have blood tests now and again (FG1-Service user5)*
Improving patient engagement with services	*And it's no problem to have a blood test. It's from 8:00 in the morning until 5:00, and you don't have an appointment, you just go (FG1-Service user5)*<br><br>*I'm lucky because my doctors know my illness. They know I've got paranoia psychosis and they know I can't do early morning, so they make me appointments for later in the daytime so they know I can be up and ready to go by that time (FG12-Service user4)*<br><br>*I've been lucky with my GP; she's very good at giving you extra time. Not allowing you to go before you've actually got your… If she finds any mental illness or any terrible illness, that it takes some time to actually voice your concerns. It might sound woolly to other people, but to you it's very important (FG4-Service user1)*<br><br>*If I want to get someone in....I'd keep picking up the phone until I got through to them. (FG5-GP4) (GP3).Then I've written to the patient and the patient hasn't attended, I've lost appointments, and I've ended up phoning them up and saying, 'Please come in,' and it takes quite a lot of effort....the personal contact definitely helps (FG5-GP4)*<br><br>*They could do drop-in sessions, like how we run our medication clinics. Just give people dates, 'Come on this date between 9:00 and 12:00, just turn up' (FG11- Community psychiatric nurse5)*<br><br>*I think also sending out regular appointments as well, because I think often if they've got an appointment, I think that makes a big difference. They do try and attend, because they've got it in writing, it's there, and again a phone call, the same (FG2-Practice nurse1)*

Category/*Sub theme*	Supporting Quotations
**Strategies to facilitate CVD risk management in SMI patients**	
Improving patient engagement with services	*Having the appointment in your diary, but maybe the surgery can remind you as well (FG8-Service user5)*<br><br>*I think GP surgeries tend to be much more rigid about how you contact people and their contact with individuals. It's the rigid appointments and you're invited by a letter, you have to make appointments through this means, and I think they may have to be more flexible than that, or more creative (FG14-Occupational therapist5)*<br><br>*Actually, I'm lucky because my doctors know my illness. They know I've got paranoia psychosis and they know I can't do early morning, so they make me appointments for later in the daytime so they know I can be up and ready to go by that time (FG12-Service user4)*<br><br>*I ask nicely if I can ring them. If you don't come because you're having a bad day, particularly some of the schizophrenic, you know, if you're having a bad day, 'Is it okay if I ring you?' They quite like that, that you can ring them (FG2-Practice nurse3)*
Continuity of care	*If you want to see a particular Dr, sometimes you might have to wait a week, but I tend to prefer to see the same Dr because then you don't have to tell your story over and over and over again (FG12-Service user3)*<br><br>*It's generally a Dr that I don't even know, and I just find those appointments are wasteful for their time and mine, because if they don't know me... it just doesn't tend to go right. That could be my fault, I'm quite happy to admit that. But it's better if I see somebody I know (FG7-Service user5)*<br><br>*Sometimes if you're in different kinds of moods, it's very hard to get to know that person because you haven't seen them before. Sometimes it takes me ages to really get to know a person and trust (FG12-Service user5)*<br><br>*I think if you've got a GP that's interested in mental health that's quite good. In our practice, we've only got two GPs – one prefers to do mental health, so he sends them all there. It's quite good because they have continuity of care with that GP as well (FG2-Practice nurse4)*<br><br>*And they trust you, and that's a big issue, isn't it, as well. I think if they trust you, then they're more likely to respect you and listen to what you're saying (FG6-Practice nurse3)*<br><br>*It comes back again to relationship with the people medically that are treating you. It helps to have a good relationship in the sense*

Category/*Sub theme*	Supporting Quotations
**Strategies to facilitate CVD risk management in SMI patients**	
Continuity of care	*that they'll go your way, in the sense if you want the information, they'll give it to you, and if you don't, you know. And still doing their job efficiently, medically at the same time, not giving you Cyanide or Penicillin when you're allergic to it (FG1-Service user)*<br><br>*You don't want to lose them; if you're too forceful you won't see them again. And that's the thing we've got to... you have to build up that relationship. (FG13-Practice nurse2)*<br><br>*You would have one person, like your healthcare assistant, who is the person who coordinates and is a point of reference within the team and where confidence could abide (FG10-GP5)*<br><br>*We try and get two permanent staff to have a joint consultation so they have always got someone they can come back to that they know then we put a note, like an alert message on the front of their sheet that they can book an appointment with this person and this person. So we almost work as buddies, particularly for SMI, so when they come in they don't have to feel they're seeing a complete stranger because that unsettles them (FG10-Practice nurse4)*<br><br>*Prior knowledge of them and their family. I think the more you know them, the more likely they are to adhere to what you say, or at least listen to what you say (FG5-GP7)*<br><br>*I think consistency, having the same person when possible. Say if they've got two practice nurses, make sure that they see the same one so they build a good rapport (FG11-Community psychiatric nurse2)*<br><br>*I think for mental health, patients with severe mental health problems, stability and trust and seeing the same person, having continuity of care is so important (FG10-GP7)*<br><br>*I think for the patient and for the nurse, actually – if you were seeing a consistent person, they'd get a bit more continuity. Then you get to know the patients better. It's like you see people for chronic disease, it's exactly the same (FG2-Practice nurse4)*<br><br>*I think the more you know them, the more likely they are to adhere to what you say, or at least listen to what you say (FG5-GP7)*<br><br>*I think patients like this would prefer to see the same person. If you start moving them around different practice nurses, they will lose that continuity, that information, you don't build up that rapport. That's one aspect, continuity of who sees them (FG6-Practice*

Category/*Sub theme*	Supporting Quotations
**Strategies to facilitate CVD risk management in SMI patients**	
Continuity of care	*nurse5)*<br><br>*And by building up that relationship, continually reviewing where they are. It's almost using that motivational interviewing technique, you know, 'Where are you today with your smoking? How do you think it affects you?' and then if you keep asking them, eventually you might get them in that loop of thinking they might give up. So I think you're right, continuity, establishing a rapport is really important (FG5-GP1)*<br><br>*I think consistency, having the same person when possible. Say if they've got two practice nurses, make sure that they see the same one so they build a good rapport (FG11-Community psychiatric nurse2)*<br><br>*The relationship is important with the patients, so people stick to somebody who is responsible for their care in a holistic way, we have a good HCA who does bloods, health promotion, and then for long term conditions the nurses are involved (FG10-GP2)*<br><br>*I think for the patient and for the nurse, actually – if you were seeing a consistent person, they'd get a bit more continuity. Then you get to know the patients better. It's like you see people for chronic disease, it's exactly the same (FG2-Practice nurse4)*
Providing positive feedback in consultations	*It's how you present it because if you say to some(one), 'You should be doing this, and you should be doing that,' uh-oh. (instead), 'Why don't you try this and why don't you try that? (FG9-Carer6)*<br><br>*I think the main thing is, 'When would you like to see me again?' not, 'I'm seeing you in three months,' Just nice and gentle (FG12-Service user2)*<br><br>*The practice nurse supported me in that I said I was doing it and she thought that was great. So I got that kind of support (FG4-Service user2)*<br><br>*"Don't do that" is a big incentive to go and do it (FG1-Service user1)*<br><br>*It's much easier giving a, 'Thou shalt...' message than a, 'Thou shalt not...' message. It's much easier saying, 'Go for a walk in the park,' than it is saying, 'Give up smoking,' or, 'Change your diet and eat much less.' It's also much easier to be successful, so in terms of positive feedback, it's much easier. All you have to do is go for a walk in the park and you've succeeded. To give up smoking, all you have to do is have one cigarette and you're a failure (FG10-GP5)*

Category/*Sub theme*	Supporting Quotations
**Strategies to facilitate CVD risk management in SMI patients**	
Providing positive feedback in consultations	*In my experience, if people don't turn up, it's not to be judgmental, and not to tell them off that they haven't arrived, but just each time they come in say, 'Oh, you didn't come last time. How about if we can do that today?' or just encourage them to keep coming, as you would with really anybody that was a poor attendee (FG2-Practice nurse4)*<br><br>*I think it's how you bill it, how you sell it. That might be... certain people would run a mile, of course, but to the vast majority of people it's like, 'Oh, people give a damn about me. People want to make sure that I don't drop dead of a heart attack as well as having schizophrenia' (FG14-Clinical psychologist2)*<br><br>*Positively reinforce messages at every visit "Well done" "25% initially, then 50%" (FG9-Carer3)*<br><br>*I think the thing is when you do get a good practice nurse, or good doctors, who actually talk to you, talk about what you're doing, it makes you realise that you're doing okay. If you know you can actually talk to them at any time, it takes you out to just realise some of the crutches you have you can deal without. So it's a subtle change. So the talking and listening aspect which I think in some cases GPs don't have the time to do (FG4-Service user1)*<br><br>*One patient, he generally doesn't want to go and see anybody else for his annual review, because he knows that I just don't go there with any of his stuff, and I don't challenge him or question him, ask him too many interrogatory questions (FG6-Practice nurse2)*<br><br>*Particularly smoking cessation, just because it didn't work that time, you try it again, and then maybe one day it will work. It's just maybe not at the time, so just don't give up. So being persistent, even though you've talked about it three months ago, when you have another screening, 'Can we talk about this again? I know you're not in that place at the moment,' so it's always spoken about so it's something we can talk about (FG11-Community psychiatric nurse2)*<br><br>*A GP is going to say, 'Right, you shouldn't smoke,' whereas a nurse is saying, 'Right, here's the plan, you have the option of taking these substitutes if you want to. You might not succeed the first time, but you might the second,' and so on and so forth (FG8-Service user2)*
Goal Setting	*I like the idea of small achievable goals (FG1-Service user1)*<br><br>*Your goals and directives would be very useful. And it's a clear structure then that you can follow and understand. Then presumably if you need to liaise with the nurse over it again, you can, or if there need to be changes made you can do that (FG8-*

Category/*Sub theme*	Supporting Quotations
**Strategies to facilitate CVD risk management in SMI patients**	
Goal Setting	*Service user2)*<br><br>*Targeting just one factor, you'll probably find that having targeted the smoking you'll find it a lot easier to get active and things like that. It's a knock on effect. Whereas if you go in, for some people with mental health problems, they want to do it all. (FG4-Service user1)*<br><br>*Identify one key behaviour change goal with the service user (FG14-Psychiatrist3)*<br><br>*Identifying one key behaviour change, it's really important to give them achievable goals and be realistic (FG6- Practice nurse1)*<br><br>*I think sometimes these small steps can make you have a bit more self-respect and look after yourself better (FG12-Service user1)*<br><br>*Looking at part goals rather than stopping smoking or getting down to an ideal BMI but making some gains in that direction is useful (FG3-GP3)*<br><br>*I think the idea of just identifying one key behaviour is a good idea, because obviously sometimes people can feel overwhelmed, can't they, by all this, so perhaps once they've realised they can do one thing and perhaps get that goal sorted, then perhaps they can then look at something else (FG11-Community Psychiatric nurse4)*
